# Dimensionality of the Chinese CES-D: Is It Stable across Gender, Time, and Samples?

**DOI:** 10.3390/ijerph182211818

**Published:** 2021-11-11

**Authors:** Diya Dou, Daniel T. L. Shek, Xiaoqin Zhu, Li Zhao

**Affiliations:** 1 Department of Applied Social Sciences, The Hong Kong Polytechnic University, Hong Kong, China; diya.dou@polyu.edu.hk (D.D.); xiaoqin.zhu@polyu.edu.hk (X.Z.); 2 West China School of Public Health/West China Fourth Hospital, Sichuan University, Chengdu 610041, China; zhaoli@scu.edu.cn

**Keywords:** depression, somatization, Chinese adolescents, factorial invariance, longitudinal invariance, replication

## Abstract

Depression is a common mental illness among Chinese adolescents. Although the Epidemiological Studies Depression Scale (CES-D) has been widely used in diverse populations, the reported factor structures are inconsistent, and its longitudinal invariance is under-researched. This study examined the psychometric properties and factorial invariance across gender and time of the CES-D among Chinese adolescents. Adolescents aged above 11 years from five schools in Chengdu responded to a questionnaire at Wave 1 (*n* = 5690). Among them, 4981 participants completed the same questionnaire after six months (Wave 2). The matched sample was composed of 4922 students (51.5% were girls; mean age = 13.15 years) at Wave 1. We used exploratory factor analysis (EFA) and confirmatory factor analysis (CFA) to examine the factor structure and performed multi-group CFA to test the factorial invariance across gender and time. A three-factor solution was identified, including “positive affect”, “somatic complaints”, and “depressed affect”. Results of multi-group CFA comparisons supported the factorial invariance of the resultant three-factor solution. Using a new sample of Chinese adolescents in Southwestern China, the present study reproduced earlier findings on adolescents in other areas in China. This study has implications for depression assessment and research in Chinese adolescents.

## 1. Introduction

Depression is a pervasive mental health issue among Chinese adolescents. A meta-analysis reviewing 51 studies with 144,060 adolescents in mainland China showed an estimated prevalence rate of depression of 24.3% [1]. Li et al.’s meta-analysis study [2] involving more than 232,000 Chinese children and adolescents also suggested that this prevalence rate had generally increased during the past three decades. Given that depression is a common cause of adolescent suicide, early diagnosis and appropriate treatment are vitally needed to reduce the suicidal risk of adolescents with depressive symptoms.

Compared with Western populations, Chinese people might conceive and manifest depression differently [3]. Previous research has consistently observed that Chinese people with mental disorders tend to report more somatic symptoms than their Western counterparts. For example, a comparative study conducted by Ryder et al. [4] found that Chinese people reported a higher frequency of somatic complaints, such as fatigue and aches, while Western samples, including American and Canadian participants, tended to express more psychological distress and emotions. Parker and colleagues’ review [5] also suggested that Chinese people tended to deny depressive feelings and express somatic complaints instead. As to Chinese adolescents, Cheng et al.’s study [6] on 11,153 Chinese high school students revealed that 12.1% of the participants tended to express psychological distress in terms of somatic symptoms. This calls for further examination of population- or culture-specific characteristics in demonstrating depression.

The 20-item CES-D (Center for Epidemiologic Studies Depression Scale) developed by Radloff [7] is a self-report measure that has been widely used to assess depressive symptoms in different populations. The CES-D indexes the frequency of 16 negative depression symptoms (e.g., “I felt depressed”) and four positive items (e.g., “I enjoyed life”) on a 4-point Likert scale. The original scale consists of four dimensions, including depressed affect (seven items, e.g., lonely and sad feelings), positive affect (four items about hopeful, happy, and enjoyable experiences), somatic and retarded activity (seven items, e.g., bothered feelings and sleep problems), and interpersonal problems (two items regarding unfriendly and dislike perceptions). This original scale was widely used in different populations in various cultures [8,9,10,11], including Chinese adolescents [3,12,13,14]. The scale possessed good psychometric properties in Chinese studies (e.g., Cronbach’s alpha = 0.87, see [12]).

However, there is controversy on the dimensionality of the CES-D. Different factor structures have been identified in studies using various samples with different languages, cultures, and depression statuses [11]. For example, Leykin et al. [11] revealed a two-factor solution with positive and negative factors based on an English sample (*n* = 3820) and a three-factor solution (i.e., somatic, positive, and negative factors) based on a Spanish sample (*n* = 13,629). With reference to the Chinese population, Wang et al. [14] found a three-factor model including “positive affect”, “somatic complaints”, and “depressed affect” based on 5059 mainland Chinese students, which was later supported by Jiang et al. [12] and Zhu et al. [15]. Alternatively, Yang, et al. [16] identified another three-factor model, which included “positive affect”, “interpersonal problem”, and “depressed affect and somatic complaints”, based on a mainland Chinese sample in rural areas. These inconsistent factor structures revealed in the literature call for further studies.

Another issue concerns the severe lack of studies on the longitudinal factorial invariance of the CES-D in Chinese populations. This issue is vital for studies on lifespan development because it addresses the basic question of whether the measure consistently reflects the same construct over time [17]. This test also clarifies whether the change in CES-D scores over time is due to fluctuation in the scale properties or genuine developmental changes [18,19]. However, research examining the longitudinal factorial invariance of CES-D remains very rare and mainly involves Western samples [20,21]. To our knowledge, only Zhu et al.’s recent study [15] tested the longitudinal factorial invariance of the CES-D using a Chinese sample over a one-year period. As such, this issue is poorly understood.

The third issue involves a lack of replication studies of longitudinal factorial invariance of measurements. Replication studies are necessary for scientific research because they can set up the basis for generalization, identify potential biases in the original research, confirm or challenge prior findings, and connect existing and new knowledge [22]. In the area of assessment, Iso-Ahola [23] highlighted the importance of replications on factorial invariance because the acceptance of “scientific truth” is significantly shaped by measurements. However, despite the importance of replication, there are very few replication studies, particularly in social sciences [24]. As to the CES-D, the analyses using data collected from a new and independent sample are considered “true replication” [25], which provides useful insight into the depression assessment in specific populations.

The present study examined the psychometric properties of the CES-D with particular reference to the replication of findings reported in Zhu et al. [15] on factorial invariance across gender and time in another adolescent sample. Based on previous findings [21], we hypothesized that the data would demonstrate the same factor structure of the CES-D as the findings in Zhu et al.’s research [15] and factorial invariance between girls and boys and over six months.

## 2. Materials and Methods

### 2.1. Participants

This study was a part of a project examining Chinese adolescent health and development in Chengdu, China, with students recruited from five schools. The five schools included primary and junior secondary schools (i.e., from grade 1 to grade 9). The first data collection (Wave 1) took place at the end of 2019 before the outbreak of the COVID-19, and the second (Wave 2) was in June and July 2020 after the resumption of face-to-face schooling. The students were invited to respond to a questionnaire in their classrooms during school hours. In total, 5690 and 4981 students aged above 11 years completed the questionnaire at Wave 1 and Wave 2, respectively. The matched sample included 4922 students (51.5% were girls) with an average age of 13.15 ± 1.32 years at Wave 1. The attrition analyses revealed no significant differences between the matched sample and the dropouts in age and gender. Except for a few students who were in grade 3 or 4, more than 99% of them were in grade 5 or above. This project was reviewed and approved by the Human Subjects Ethics Subcommittee at the authors’ university. Written informed consent was gained from school principals, students, and parents before the data collection.

### 2.2. Measures

A Chinese version of Radloff’s 20-item CES-D scale [7] was adopted in the present study. This scale has been widely used and validated in previous studies in Chinese populations [14,15,26,27]. Students reported the frequency of each symptom they experienced in the past seven days. A 4-point Likert scale was used (0 = “Rarely or less than 1 day”, 1 = “Some of the time or 1–2 days”, 2 = “A moderate amount of the time or 3–4 days”, and 3 = “Most or all of the time or 5–7 days”). The four items pertaining to positive perceptions were reverse-coded.

### 2.3. Data Analysis

Following some scholars’ suggestions [14,20,28], we first conducted exploratory factor analysis (EFA), then performed confirmatory factor analysis (CFA), and lastly tested factorial invariance by gender and over time. The full dataset collected at Wave 1 (*n* = 5690) was randomly divided into two subsamples. We performed EFA on subsample A (*n* = 2845) using principal components analysis (PCA) with promax rotation, as suggested in previous studies [12,14]. Pairwise deletion was used to treat missing data. SPSS Version 26.0 (IBM Corp: Armonk, NY, USA) was used to handle data and perform EFA.

We further conducted CFA using subsample B (*n* = 2845) to determine the final factor structure. In total, five models were tested and compared (see Table 1), including Radloff’s original 4-factor model (Model 1); a two-factor model distinguishing positive items from the negative ones [11,13,29] (Model 2); a three-factor model including “positive affect”, “somatic complaints”, and “depressed affect” proposed by Wang et al. [14] (Model 3); a three-factor model containing “positive affect”, “interpersonal problem”, and one factor combining the original “depressed affect” and “somatic complaints” identified by Yang et al. [16] (Model 4); and the model based on previous EFA results [15] (Model 5).

For the CFAs, the fit indices included chi-square (*χ*^2^), comparative fit index (CFI), Tucker–Lewis index (TLI), and root mean square error of approximation (RMSEA). In general, CFI and TLI larger than 0.90 and RMSEA smaller than 0.080 were commonly used cutoff criteria [30]. For model comparison, the Bayesian information criterion (BIC) was used as an indicator. According to Raftery [31], a decrease in BIC suggests a better model fit, and a ΔBIC value larger than 10 indicates a satisfactory model improvement. Mplus Version 8.5 was used to conduct CFA. Considering that the CES-D used a 4-point Likert scale, which should be regarded as categorical data rather than continuous data [32], we used weighted least squares means and variance adjusted estimator (WLMSV), as it is recommended for modeling categorical or ordered data [33].

Based on the CFA findings, we tested measurement invariance by gender at two waves (Wave 1: *n* = 5690, Wave 2: *n* = 4981) and across time using the full matched sample of two waves (*n* = 4922). Following the procedure widely adopted in previous studies [34,35], we conducted a series of measurement invariance tests, including (1) a configural invariance test that allows parameters to differ across groups; (2) a metric invariance test, which assumes equal factor loadings across groups; (3) a scalar invariance test that adds constraints of equal intercepts across groups, and (4) a strict invariance model, which further forces factor variances and covariances to be equal between groups. In addition, we constrained latent means across groups to explore potential gender differences in the level of depression. The indices for model comparisons included the absolute value of the changes in CFI (|∆CFI|) with a cutoff value < 0.01 and that in RMSEA (|∆RMSEA|) with a cutoff value < 0.015 [36,37]. Mplus Version 8.5 was used to conduct CFA and factorial invariance tests.

Similar analyses were performed to test measurement invariance over time. Again, we established and compared the nested models, including configural, metric, scalar, and strict invariance models based on the full dataset at Wave 1 and Wave 2, respectively. The parameters were constrained equally across the two groups (i.e., Wave 1 and Wave 2 data). Latent mean comparisons were conducted to examine the change in the level of depression over time. The same indices and criteria were adopted (|∆CFI| < 0.01 and |∆RMSEA| < 0.015) [36,37].

## 3. Results

### 3.1. Exploratory Factor Analysis

As shown in Table 2, results of EFA retained three factors with eigenvalues greater than 1.0, which explained 39.40%, 11.79%, and 5.77% of the total variance, respectively. A new “somatic complaints” factor was identified, which contained six items (Items 1, 2, 5, 7, 11, and 20) originally loaded on the “somatic complaints” and four items (Items 3, 6, 9, and 10) originally under “depressed affect” of Radloff’s original model. Next, a new “depressed affect” factor included three original “depressed affect” items (Items 14, 17, and 18), two original “interpersonal problem” items (Item 15 and 19), and one “somatic complaints” item (Item 13) in Radloff’s model. The third factor consisted of the four “positive affect” items (Items 4, 8, 12, and 16).

Similar to many previous findings based on Chinese populations [14,38], the three-factor solution revealed by the EFA results was inconsistent with Radloff’s original proposed structure. The original “interpersonal problem” factor was not identified, while the related items under this factor loaded on the “depressed affect” factor. In addition, the new “somatic complaints” factor covered three items that were originally under “depressed affect.”

### 3.2. Confirmatory Factor Analysis and Model Comparisons

Table 3 summarizes the results of CFA and model comparisons. Among the five competing models with different factor structures, Models 1, 2, and 4 demonstrated less satisfactory model fit, with RMSEAs over 0.08. Model 3 and Model 5 showed acceptable model fit, with CFIs and TLIs over 0.90 and RMSEAs below 0.08. As compared to Model 3, Model 5 demonstrated a lower BIC value (ΔBIC = 28.30 > 10), suggesting a better model fit. Therefore, Model 5 was identified as the model that our data fit the best (WLSMV *χ*^2^(167) = 2918.995, CFI = 0.951, TLI = 0.944, RMSEA = 0.076 (90% CI: 0.074, 0.079), BIC = 124199.661).

### 3.3. Measurement Invariance across Gender

Based on the results of CFA, Model 5 was retained for measurement invariance tests. First, we tested the fit of Model 5 for boys and girls separately. Results showed that the tested model indicated a good fit for both groups, with CFIs > 0.90, TLIs > 0.90, and RMSEAs < 0.08 (see Table 4). Second, we compared the fit of each pair of the nested models (i.e., configural, metric, scalar, and strict invariance models). The absolute values of ∆CFIs (<0.01) and ∆RMSEAs (<0.015) also supported the configural, metric, scalar, and strict invariance of the factor structure between girls and boys at Wave 1 and Wave 2 (see Table 4). In addition, the results of the latent mean invariance test also suggested the fit indices between boys and girls were equivalent, with |∆CFI| lower than 0.01 and |∆RMSEA| below 0.015 (see Table 4). When the latent means of the three factors were forced to be zero for boys, the latent means of “somatic complaints”, “depressed affect”, and “positive affect” for girls were 0.163, 0.113, and –0.059 (*p*s < 0.01) at Wave 1 and were 0.196, 0.031, and –0.092 (*p*s < 0.001) at Wave 2, respectively. Additionally, the standardized effect sizes of gender differences of the three factors were 0.187, 0.169, and 0.087 at Wave 1 and were 0.214, 0.201, and 0.102 at Wave 2, respectively. The results suggested gender differences of the three factors were small [35,39].

### 3.4. Measurement Invariance over Time

The resultant three-factor model demonstrated acceptable model fit for Wave 1 data (WLSMV *χ*^2^(167) = 4419.322, CFI = 0.954, TLI = 0.947, RMSEA = 0.072, see Table 4). For Wave 2 data, the model demonstrated acceptable CFI and TLI values larger than 0.90, but the RMSEA value was 0.082, which was slightly above the cut-off value. Results showed that the metric, scalar, and strict longitudinal invariance were supported (|∆CFI| < 0.01, |∆RMSEA| < 0.015, see Table 4). Generally speaking, the findings supported the longitudinal invariance of the three-factor model of the CES-D over six months.

Standardized factor loadings of all items, average inter-item correlations, composite reliability (CR), and average extracted variance (AVE) are summarized in Table 5. The average inter-item correlations were greater than 0.4, the coefficient alpha values were above 0.7, and composite reliability was above 0.8 for all factors at both waves, demonstrating good internal consistency. The scale demonstrated good test-retest reliability (ICC = 0.712, 95% CI: 0.695, 0.728) over a six-month period. AVEs were above 0.5 for all the factors at two waves, denoting that the latent variables accounted for more than 50% of the variance of observed items and thus showing satisfactory construct validity over time [40]. Finally, results of the longitudinal latent mean invariance test also suggested a negligible difference in the three factors between the two waves (|∆CFI| < 0.01, |∆RMSEA| < 0.015, see Table 4). After fixing the latent means for three factors of Wave 1 data to zero, the latent means of “somatic complaints”, “depressed affect”, and “positive affect” at Wave 2 were 0.110, 0.051, and 0.063 (*p*s < 0.001), respectively. Additionally, the standardized effect sizes of the differences were 0.121, 0.078, and 0.082, respectively. As the effect sizes were small, the longitudinal stability of the CES-D scores over six months was supported.

## 4. Discussion

This study examined the dimensionality and factorial invariance of the CES-D using two-wave data collected from 4922 Chinese adolescents over six months. Compared to previous studies, this study has some strengths. First, the sample size of the present study was relatively large. Second, by adopting a sample of Chinese adolescents, this research responded to the call for exploration of depression in non-Western societies rather than taking the Western respondents as the baseline from which other populations may deviate, which is consistent with the argument that how people conceive and express depression should be interpreted through the specific cultural lens [4,5]. As the largest ethnic group, the Chinese account for one-fifth of the global population. The instruments developed and widely used in Western literature do not necessarily suggest the same norm for depression among Chinese adolescents. Third, our research added to the limited inquiry on the longitudinal validity of the CES-D. The assumption that the measures consistently reflect the same construct over time should be tested to warrant the accuracy of the models aiming to reveal developmental changes [17,41]. Fourth, this study is a replication study of Zhu et al.’s research [15] on 2648 Chinese students in other urban areas in China. This study used a new sample of 4922 adolescents in Southwestern China (Sichuan province), and many participants lived in less developed or rural areas. The results provided further evidence on the factor structure and invariance of the CES-D among Chinese adolescents with relatively lower levels of socioeconomic status.

This study replicated the findings reported in Zhu et al.’s research [15] that there are three factors in the CES-D, including “positive affect”, “somatic complaints”, and “depressed affect.” In particular, the “interpersonal problem” factor in Radloff’s model did not emerge, and the corresponding items appeared under the “depressed affection” dimension. In addition, some items originally under “depressed affect” were found to reflect “somatic complaints” in our study. Although inconsistent with Radloff’s original one, our results are in line with previous evidence drawn from Chinese populations [14,38]. In addition, our results indicated that the measure reflected the same construct for boys and girls and showed longitudinal stability over six months, which also reproduced Zhu et al.’s findings [15].

This study revealed Chinese adolescents’ tendency to express depression through somatic symptoms instead of psychological distress symptoms. This unique conception of depression has been consistently observed in other empirical studies in Chinese adult samples [3,4,5] and adolescent samples [6]. This observation can be explained in terms of the argument that somatization is more acceptable for expressing distress by the Chinese culture, which is precisely described in the Chinese proverb that “man can shed blood but not tears” [42]. Tung [43] proposed a linguistic explanation that many Chinese terms describing negative emotional experience are related to body pain, and thus, the Chinese population may spontaneously use somatic descriptors to express depressed emotions. Additionally, somatization is often observed among adolescents [6]. This may be due to the fact that adolescents’ abilities to verbalize emotional distress are not yet fully developed. Moreover, previous work has revealed the existence of mental health stigma in Chinese societies [44,45], which may result in the tendency to somatize psychological distress among Chinese adolescents due to negative public labeling.

Given that different conceptions lead to specific depression symptoms profiles, diagnosis, and treatments, our findings have clinical implications. First, Chinese adolescents may report a lower level of depression due to masking depressed feelings (e.g., sadness) with somatic complaints (e.g., insomnia). Second, in addition to screening tools, such as the CES-D, follow-up questions and probes would be helpful to uncover depressed symptoms of Chinese adolescents [4].

Results supported that the CES-D was longitudinally invariant in a sample of Chinese adolescents over six months. The longitudinal invariance of measurement is fundamental for developmental research. The investigation on developmental change should be established on the assumption that the repeatedly measured variables could reflect the same constructs over time [46]. Our findings gauged the longitudinal invariance of the CES-D, which would allow researchers to use this scale to signal true changes and model developmental patterns of depression during adolescence when youth experience an increase in depressive symptoms and have more needs for related education and intervention. Moreover, it is worth noting that the first data collection took place before the outbreak of the COVID-19, while the second data collection was conducted six months later, when the severity of COVID-19 has gradually reduced in China. Our findings suggest that, with the change in time in which major events happened, the factor structure of CES-D remained stable.

This study highlighted the importance of replication of depression assessment findings. As discussed earlier, replication serves as the foundation of scientific knowledge accumulation [47]. For studies on measurement invariance, replication can retest the assumptions required by statistics methods that may evolve over time and differ among populations [48]. Moreover, replication studies on measurement invariance are particularly important for cross-cultural studies, as these studies ensure that the same constructs are assessed in all cultural groups [4]. Successful replication studies provide support for measurement validity, which would help researchers draw robust explanations for phenomena that were assessed by latent constructs. As a replication study of Zhu et al.’s research [15], this study corroborated the original findings that the three-factor solution of the CES-D demonstrated robustness across gender and over time. This study responded to the ongoing call for the accumulation of replication data using new statistics methods [47]. We also join the appeal that more replication studies should be conducted in psychology and psychiatry research as a routine practice [48].

This study has some limitations. First, this study used two waves of data across six months. Future analysis will benefit from longitudinal studies involving more than two measurement waves and a longer time duration between successive waves of data collection. With multiple waves of data, additional constraints could be added to the model to test the stability of the cross-lagged covariances, which could serve as an invariance model at a stricter level [46]. Second, this study only used an adolescent sample in one province in China. Future studies may recruit participants in other Chinese societies. Given that the Chinese are the largest ethnic group in the world, it is problematic to assume that this large population is homogeneous [5]. For example, adolescents in Hong Kong or Macau may be strongly influenced by a mix of Eastern and Western cultures and thus conceive and express distress feelings differently [13]. This will provide insights to develop culturally sensible measurements and establish equivalence based on samples from different Chinese societies.

## 5. Conclusions

By replicating Zhu et al.’s work [15], this study is a positive response to the call for establishing measurement equivalence of the CES-D among adolescents in non-Western cultures [14]. This study reproduced the original results with the identification of the same three-factor structure and factorial invariance across gender and over time. This study highlights the importance of replication research in psychology and psychiatry and has implications for depression assessment for Chinese adolescents.

## Figures and Tables

**Table 1:** Item mapping for tested competing models.

No	Item Content	Model 1	Model 2	Model 3	Model 4	Model 5
1	I was bothered by things that usually don’t bother me	SC	DA	SC	DA	SC
2	My appetite was poor	SC	DA	SC	DA	SC
3	I felt that I could not shake off the blues even with help from my family or friends	DA	DA	SC	DA	SC
4	I felt I was just as good as others	PA	PA	PA	PA	PA
5	I had trouble keeping my mind on what I was doing	SC	DA	SC	DA	SC
6	I felt depressed	DA	DA	SC	DA	SC
7	I felt that everything I did was an effort	SC	DA	SC	DA	SC
8	I felt hopeful about the future	PA	PA	PA	PA	PA
9	I thought my life had been a failure	DA	DA	SC	DA	SC
10	I was fearful	DA	DA	SC	DA	SC
11	My sleep was restless	SC	DA	SC	DA	SC
12	I was happy	PA	PA	PA	PA	PA
13	I talked less than usual	SC	DA	DA	DA	DA
14	I felt lonely	DA	DA	DA	DA	DA
15	People were unfriendly	IP	DA	DA	IP	DA
16	I enjoyed life	PA	PA	PA	PA	PA
17	I had crying spells	DA	DA	DA	DA	DA
18	I felt sad	DA	DA	DA	DA	DA
19	I felt that people disliked me	IP	DA	DA	IP	DA
20	I could not get “going”	SC	DA	DA	DA	SC

Note. DA, depressed affect; IP, interpersonal problem; PA, positive affect; SC, somatic complaints; Model 1, Radloff’s original four-factor model; Model 2, a two-factor model in which all negative items were combined into an independent factor, and the remaining four positive items formed a second factor; Model 3, a three-factor model in which positive affect and two new factors merged from original depressed affect, interpersonal problem, and somatic complaints; Model 4, another three-factor model with positive affect, interpersonal problem, and a new depressed affect factor including original depressed affect and somatic complaints; Model 5, the three-factor model identified in the authors’ previous work and in the present exploratory factor analysis (EFA).

**Table 2:** Factor loadings for exploratory factor analysis using the half sample A at Wave 1 (*n* = 2845).

No	Item Content	SC	DA	PA
1	I was bothered by things that usually don’t bother me	**0.636**	0.180	0.004
2	My appetite was poor	**0.576**	0.127	−0.027
3	I felt that I could not shake off the blues even with help from my family or friends	**0.653**	0.261	0.062
4	I felt I was just as good as others	−0.076	0.099	**0.725**
5	I had trouble keeping my mind on what I was doing	**0.744**	0.181	0.005
6	I felt depressed	**0.752**	0.334	0.120
7	I felt that everything I did was an effort	**0.762**	0.291	0.091
8	I felt hopeful about the future	−0.063	−0.010	**0.728**
9	I thought my life had been a failure	**0.532**	0.429	0.099
10	I was fearful	**0.588**	0.424	0.074
11	My sleep was restless	**0.591**	0.305	0.031
12	I was happy	0.206	0.031	**0.819**
13	I talked less than usual	0.312	**0.440**	−0.153
14	I felt lonely	0.249	**0.755**	0.088
15	People were unfriendly	0.191	**0.803**	0.040
16	I enjoyed life	0.171	0.091	**0.819**
17	I had crying spells	0.454	**0.621**	0.116
18	I felt sad	0.468	**0.659**	0.102
19	I felt that people disliked me	0.311	**0.773**	0.100
20	I could not get “going”	**0.587**	0.490	0.101
Explained variance		39.40%	11.79%	5.77%

Note. SC, somatic complaints; DA, depressed affect; PA, positive affect. Cronbach’s alphas were 0.894, 0.834, and 0.786 for SC, DA, and PA, respectively. The highest factor loading on the three factors of each item is highlighted in bold.

**Table 3:** Model comparisons for tested models using the half sample B at Wave 1 (*n* = 2845).

Model	WLSMV *χ*^2^	*df*	CFI	TLI	BIC	RMSEA (90% CI)
Model 1	3282.130 ***	164	0.944	0.936	124,509.874	0.082 (0.079, 0.084)
Model 2	3573.585 ***	169	0.939	0.932	125,022.618	0.084 (0.082, 0.087)
Model 3	3041.347 ***	167	0.949	0.942	124,227.959	0.078 (0.075, 0.080)
Model 4	3473.726 ***	167	0.941	0.933	124,721.648	0.083 (0.081, 0.086)
Model 5	2918.995 ***	167	0.951	0.944	124,199.661	0.076 (0.074, 0.079)

Note. WLSMV, weighted least squares with mean and variance adjustment; df, degree of freedom; CFI, comparative fit index; TLI, Tucker–Lewis index; BIC, Bayesian information criterion; RMSEA, root mean square error of approximation; CI, confidence interval; Model 1, Radloff’s original four-factor model; Model 2, a two-factor model in which all negative items were combined into an independent factor, and the remaining four positive items formed a second factor; Model 3, a three-factor model in which positive affect and two new factors merged from original depressed affect, interpersonal problem, and somatic complaints; Model 4, another three-factor model with positive affect, interpersonal problem, and a new depressed affect factor including original depressed affect and somatic complaints; Model 5, the three-factor model identified in the present exploratory factor analysis (EFA). *** *p* < 0.001.

**Table 4:** Measurement invariance tests across gender based on the full samples at Wave 1 and Wave 2 and over time based on the matched sample.

	WLSMV *χ*^2^	*df*	CFI	TLI	RMSEA (90% CI)	Comparison	Δ*χ*^2^	ΔCFI	ΔRSMEA
Invariance tests across gender at Wave 1									
Boys (*n* = 2922)	2628.394 ***	167	0.949	0.942	0.071 (0.069, 0.073)				
Girls (*n* = 2768)	2895.325 ***	167	0.959	0.953	0.077 (0.074, 0.079)				
A. Configural invariance	5506.333 ***	334	0.954	0.948	0.074 (0.072, 0.075)				
B. Metric invariance	5611.683 ***	351	0.954	0.950	0.073 (0.071, 0.074)	B vs. A	126.447 ***	0.000	−0.001
C. Scalar invariance	5285.871 ***	388	0.957	0.958	0.067 (0.065, 0.068)	C vs. B	85.472 ***	0.003	−0.006
D. Strict invariance	4327.805 ***	394	0.956	0.967	0.059 (0.058, 0.061)	D vs. C	161.467 ***	0.008	−0.008
E. Latent mean invariance	3636.503 ***	397	0.971	0.973	0.054 (0.052, 0.055)	E vs. D	47.287 ***	0.006	−0.005
Invariance tests across gender at Wave 2									
Boys (*n* = 2566)	3000.549 ***	167	0.952	0.945	0.081 (0.079, 0.084)				
Girls (*n* = 2415)	3033.410 ***	167	0.965	0.960	0.084 (0.082, 0.087)				
A. Configural invariance	6027.779 ***	334	0.959	0.954	0.083 (0.081, 0.085)				
B. Metric invariance	6174.620 ***	351	0.958	0.955	0.082 (0.080, 0.083)	B vs. A	164.064 ***	−0.001	−0.001
C. Scalar invariance	5964.862 ***	388	0.960	0.961	0.076 (0.074, 0.078)	C vs. B	114.739 ***	0.002	−0.006
D. Strict invariance	4981.181 ***	394	0.967	0.968	0.068 (0.067, 0.070)	D vs. C	245.181 ***	0.007	−0.008
E. Latent mean invariance	4283.351 ***	397	0.972	0.973	0.063 (0.061, 0.064)	E vs. D	60.211 ***	0.005	−0.007
Invariance tests over time									
Wave 1 (*n* = 4922)	4419.322 ***	167	0.954	0.947	0.072 (0.070, 0.074)				
Wave 2 (*n* = 4922)	5741.640 ***	167	0.959	0.953	0.082 (0.081, 0.084)				
A. Configural invariance	9464.917 ***	705	0.956	0.951	0.050 (0.049, 0.051)				
B. Metric invariance	9557.669 ***	722	0.955	0.952	0.050 (0.049, 0.051)	B vs. A	87.551 ***	−0.001	0.000
C. Scalar invariance	9501.125 ***	759	0.956	0.954	0.048 (0.048, 0.049)	C vs. B	103.317 ***	0.001	−0.002
D. Strict invariance	8148.139 ***	765	0.963	0.962	0.044 (0.043, 0.045)	D vs. C	14.303 *	0.007	−0.004
E. Latent mean invariance	7858.696 ***	768	0.964	0.964	0.043 (0.042, 0.044)	E vs. D	70.424 ***	0.001	−0.001

Note. WLSMV, weighted least squares with mean and variance adjustment; df, degree of freedom; CFI, comparative fit index; TLI, Tucker–Lewis index; RMSEA, root mean square error of approximation; CI, confidence interval; Δ*χ*^2^, change in chi-square (obtained from *DIFFTEST* in Mplus); ΔCFI, change in CFI; ΔRMSEA, change in RMSEA. * *p* < 0.05; *** *p* < 0.001.

**Table 5:** Standardized factor loadings and psychometric properties for the longitudinal invariance model of CES-D.

No	Item Content	Wave 1			Wave 2		
		SC	DA	PA	SC	DA	PA
1	I was bothered by things that usually don’t bother me	0.672			0.692		
2	My appetite was poor	0.596			0.656		
3	I felt that I could not shake off the blues even with help from my family or friends	0.769			0.812		
5	I had trouble keeping my mind on what I was doing	0.743			0.798		
6	I felt depressed	0.871			0.905		
7	I felt that everything I did was an effort	0.856			0.892		
9	I thought my life had been a failure	0.752			0.804		
10	I was fearful	0.791			0.843		
11	My sleep was restless	0.709			0.779		
20	I could not get “going”	0.848			0.893		
13	I talked less than usual		0.548			0.656	
14	I felt lonely		0.810			0.859	
15	People were unfriendly		0.801			0.849	
17	I had crying spells		0.879			0.919	
18	I felt sad		0.905			0.941	
19	I felt that people disliked me		0.872			0.897	
4	I felt I was just as good as others			0.610			0.682
8	I felt hopeful about the future			0.512			0.534
12	I was happy			0.903			0.911
16	I enjoyed life			0.940			0.922
	Mean factor loading	0.761	0.803	0.741	0.807	0.854	0.762
	Average variance extracted	0.585	0.658	0.583	0.658	0.737	0.608
	Composite reliability	0.933	0.919	0.841	0.950	0.943	0.856
	Cronbach’s α	0.890	0.847	0.783	0.914	0.887	0.809
	Mean inter-item correlation	0.448	0.485	0.473	0.517	0.569	0.512

Note. CES-D, The Center for Epidemiologic Studies Depression Scale; SC, somatic complaints; DA, depressed affect; PA, positive affect.

## Data Availability

The data presented in this study are available on request from the corresponding author. The data are not publicly available due to privacy.
